# The Characterization and Antioxidant and Erythroprotective Effects of β-Carotene Complexed in β-Cyclodextrin

**DOI:** 10.3390/ijms26083902

**Published:** 2025-04-20

**Authors:** Andrés Leobardo Puebla-Duarte, Ariadna Thalía Bernal-Mercado, Irela Santos-Sauceda, Mónica Acosta-Elias, Daniel Fernández-Quiroz, Silvia Elena Burruel-Ibarra, José de Jesús Ornelas-Paz, Ingrid Daniela Pérez-Cabral, Francisco Rodríguez-Félix, Rey David Iturralde-García, Miguel Ángel Robles-García, José Agustín Tapia-Hernández, Ricardo Iván González-Vega, Carmen Lizette Del-Toro-Sánchez

**Affiliations:** 1 Departamento de Investigación y Posgrado en Alimentos, Universidad de Sonora, Blvd. Luis Encinas y Rosales S/N, Col. Centro, Hermosillo 83000, Sonora, Mexico; a215201577@unison.mx (A.L.P.-D.); a222230199@unison.mx (I.D.P.-C.); francisco.rodriguezfelix@unison.mx (F.R.-F.); rey.iturralde@unison.mx (R.D.I.-G.); joseagustin.tapia@unison.mx (J.A.T.-H.); 2 Departamento de Investigación en Polímero y Materiales, Universidad de Sonora, Blvd. Luis Encinas y Rosales S/N, Col. Centro, Hermosillo 83000, Sonora, Mexico; irela.santos@unison.mx (I.S.-S.); silvia.burruel@unison.mx (S.E.B.-I.); 3 Departamento de Investigación en Física, Universidad de Sonora, Blvd. Luis Encinas y Rosales S/N, Col. Centro, Hermosillo 83000, Sonora, Mexico; monica.acosta@unison.mx; 4 Departamento de Ingeniería Química y Metalurgia, Universidad de Sonora, Blvd. Luis Encinas y Rosales S/N, Col. Centro, Hermosillo 83000, Sonora, Mexico; daniel.fernandez@unison.mx; 5 Centro de Investigación en Alimentación y Desarrollo, A.C.-Unidad Cuauhtémoc, Av. Río Conchos S/N, Parque Industrial, Ciudad Cuauhtémoc 31570, Chihuahua, Mexico; jornelas@ciad.mx; 6 Departamento de Ciencias Médicas y de la Vida, Centro Universitario de la Ciénega, Universidad de Guadalajara, Av. Universidad 1115, Col. Lindavista, Ocotlán 47820, Jalisco, Mexico; miguel.robles@academicos.udg.mx; 7 Departamento de Ciencias de la Salud, Centro Universitario de los Valles, Universidad de Guadalajara, Carr. A Guadalajara Km 45.5., Ameca 46600, Jalisco, Mexico; ricardo.gonzalez@academicos.udg.mx

**Keywords:** beta-cyclodextrin, beta-carotene, inclusion complex, antioxidant, erythroprotective, encapsulation

## Abstract

β-carotene (β-C) is a hydrophobic compound, easily degradable by light and oxygen and with low solubility, limiting its applications. β-cyclodextrin (β-CD) can encapsulate β-C, protecting it from degradation and maintaining its bioactivity. Therefore, this research aimed to characterize and determine the antioxidant and erythroprotective activity of β-C/β-CD inclusion complexes. The co-precipitation technique was used to elaborate β-C/β-CD in a 40:60 ratio, obtaining a high yield (94.10%), an entrapment efficiency of 82.47%, and a loading efficiency of 11.92%. The moisture of β-C/β-CD was 2.93%. β-C release increased over the time of 216 h (80.8%, 92.8%, and 97.4% at 8 °C, 25 °C, and 37 °C, respectively). A UV–visible analysis confirmed the presence of β-carotene in the inclusion complex, indicating successful encapsulation without significant structural changes. According to the adsorption–desorption isotherms, the complexes showed a type II isotherm. The FT-IR and Raman spectroscopy confirmed the formation of the inclusion complex, which interacted by hydrogen bonds, hydrophobic interactions, or van der Waals forces. The DSC showed an endothermic peak at 118 °C in the β-C/β:CD. The TGA revealed reduced water loss in the β-carotene/β-cyclodextrin complex, indicating limited water binding due to encapsulation. The microscopic surface morphologies observed by the SEM of β-C/β-CD were irregular-shaped clumps in the surface with a particle average size of 8.09 µm. The X-ray diffraction showed a crystalline structure of the complex. The zeta potential determination indicated a negative charge (−23 and −32 mV). The ABTS, DPPH, and FRAP demonstrated the antioxidant activity of β-C/β:CD (34.09%, 21.73%, and 8.85. mM ET/g, respectively), similar to pure β-C (34.64%, 22.63%, and 9.12 μM ET/g, respectively). The complexes showed an erythroprotective effect inhibiting hemolysis (64.09%). Therefore, with these characteristics, β-CD is a good encapsulant for β-C, and this complex could be applied in the food and pharmaceutical industries.

## 1. Introduction

The development of promising transport mechanisms for bioactive compounds and their complications, due to their nature, are of utmost nutrimental significance in contemporary foods. Carotenoids, such as β-Carotene (β-C), are bioactive compounds that have acquired research interest for their potential antioxidant activity against radicals and health-protective properties [1,2,3]. However, the structure of β-C (a highly conjugated polyene with eleven conjugated double bonds and two β-rings) makes it singularly susceptible to oxidative degradation when exposed to light, oxygen, temperature, and pH [4]. These characteristics and the high hydrophobicity and low solubility of β-C are notable barriers to its oral formulation and bioavailability [5]. An alternative for this is its encapsulation in β-Cyclodextrin (β-CD).

β-CD comprises cyclic oligosaccharides of seven glucose molecules linked by α-1,4 D-glucopyranoside bonds. β-CD is crystalline and has a torus-shaped structure, creating a cone where its exterior surface is hydrophilic and the interior is hydrophobic [6,7]. Therefore, these properties could encapsulate β-C inside, forming inclusion complexes. According to other studies, hydrophilic molecules (oils, lipids, vitamins, and pigments, among others) can interact with β-CD through weak bonds such as hydrogen bonds, van der Waals, or hydrophobic forces. In this process, the enthalpy-rich water molecules within the internal cavity are partially or wholly displaced, while the inclusion complex is rendered thermodynamically stable [8,9,10,11,12].

These complexes are gaining interest in the food industry and drug delivery. A drug delivery system offers diverse advantages, such as being included in aqueous, solid, or semi-solid systems [13]. Recently, cyclodextrin inclusion technology has reached satisfactory results in improving the solubility, oxidative and thermal stability, controlled release, and bioavailability of bioactive compounds [14,15,16,17,18,19]. Additionally, they are not toxic because of their biodegradability and biocompatibility with the human body [6,12,20]. While liposomes and nanoparticles are highly effective for encapsulating bioactive compounds, they often require complex preparation techniques and expensive materials, and they may exhibit stability issues, particularly under storage conditions involving temperature and light fluctuations. In contrast, β-CD offers a simpler and cost-effective encapsulation approach, providing enhanced thermal and oxidative stability [21,22]. Additionally, β-CD’s unique toroidal structure allows it to form inclusion complexes with lipophilic compounds like β-C, significantly improving its solubility and bioavailability [23]. Unlike liposomes, β-CD is a solid, crystalline material that is easier to handle and incorporate into various food and pharmaceutical formulations without requiring advanced emulsification or dispersion techniques [24]. These advantages, coupled with its non-toxic, biodegradable, and biocompatible nature, make β-CD a highly suitable and practical choice for encapsulating β-C in our study.

On the other hand, when a bioactive molecule crosses the intestinal barrier, it reaches the circulation to subsequently have its action on the target tissues (bioavailability) [25]. However, in the blood, there is also the presence of radicals (reactive oxygen species) that can damage erythrocytes by oxidizing them and thus bring future damage, both in the transport of substances and in the generation of chronic degenerative diseases [26,27]. To harness the antioxidant properties of β-C, our research focuses on encapsulating it within β-CD to protect it from degradation, preserve its antioxidant activity, and evaluate its ability to inhibit the radicals that can damage erythrocytes. This approach not only achieves an erythroprotective effect but also highlights the innovative potential of this study.

## 2. Results

### 2.1. Yield, Entrapment Efficiency, and Loading Determination

In this study, the formation of the β-C/β-CD inclusion complex was evaluated based on process efficiency, including yield percentage (%), entrapment efficiency (EE%), and loading efficiency (LE%) (Table 1). The yield represents the efficiency of the formation process selected to obtain the inclusion complex through the drying technique (e.g., precipitation) [28]. The yield was 94.10 ± 1.21%, reflecting the suitability of preparing the inclusion complex by the precipitation technique. The percentage of active compound encapsulated (β-C) in the β-CD was quantified and determined (EE%), resulting in 82.47 ± 0.40%. According to the values of pure β-C “loaded” in the inclusion complex, it was determined as 35 ± 0.24%. These encapsulation parameters vary notably with the preparation method and the stoichiometric ratio used in the literature. For example, using emulsion, Durante et al. [29] obtained a 97% EE. In another study, Yazdani et al. [29] prepared β-carotene/β-cd complexes from a precipitation method, obtaining 61.46% and 24.69% of EE and LC, respectively. Thus, the values obtained in this work are close to those in the literature.

### 2.2. UV–Visible Spectra of β-Carotene, β-CD, and β-C/β-CD Inclusion Complex

**Figure 1:**
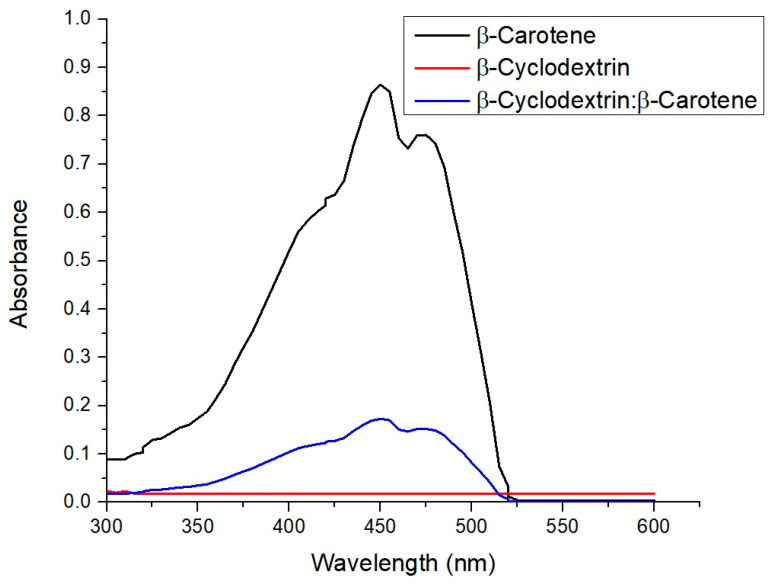
UV–visible spectra of β-carotene, β-CD, and the β-C/β-CD inclusion complex.

The UV–visible spectra of β-carotene, β-CD, and the β-C/β-CD inclusion complex are shown in Figure 1. The adsorption peak of β−C is observed at 453 nm. As can be seen, the oligosaccharide is not visible in this region. On the other hand, the complex exhibits absorption at the same wavelength as the carotenoid compound but with a significantly lower intensity. This result is consistent with the research conducted by Yazdani et al. [29], who reported the production of inclusion complexes from different cyclodextrin derivatives with β-carotene. Other studies have reported that inclusion complex formation produces a hypsochromic shift in the absorption maxima of β-C [30]; however, this phenomenon is not observed in our work.

### 2.3. Moisture Content

The efficiency of drying, or the moisture content, is a significant parameter in assessing powder quality and has a notable impact on various aspects, including technological properties, shelf life, and powder packaging. It plays a crucial role in determining the stability of a powder. The results showed that, for pure β-CD, a value of 13% of humidity was obtained. However, when the β-C was complexed with β-CD, the humidity was lower, obtaining 2.93%.

### 2.4. Determination of Release Profiles

**Figure 2:**
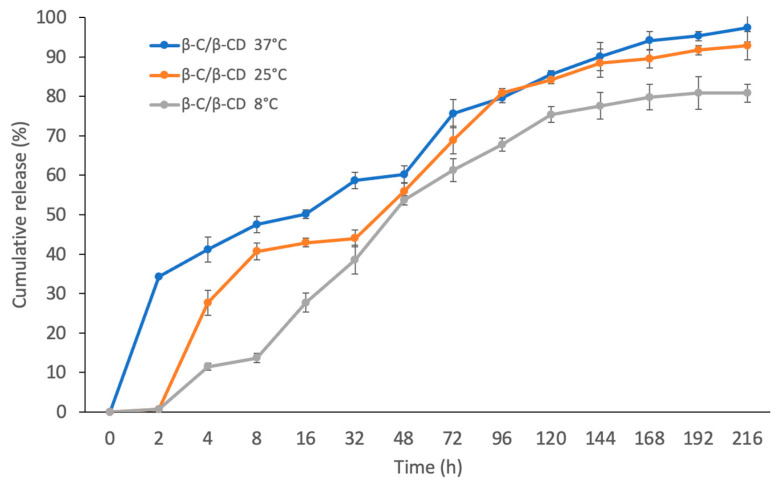
Cumulative release rate of the β-C for the β-/β-CD inclusion complex at temperatures of 8, 25, and 37 °C.

The release of β-C from the β-C/β-CD inclusion complex was studied at 8, 25, and 37 °C. The in vitro release experiments showed that the β-C release had a similar profile at the three different temperature values (Figure 2). However, it is worth mentioning that a slightly higher release rate of β-C was observed at 37 °C (97.4%) compared to at 8 °C (80.8%) and 25 °C (92.8%).

The release profile of the β-C/β-CD inclusion complex demonstrated a rapid release effect at 2 h at 37 °C with a release rate of 34.25%, while at 4 h the release began at 8 °C and 25 °C (11.5% and 27.7%, respectively). This may be attributed to the release of the β-C weakly bound to the surface of the β-CD [31,32]. A rapid rise was gently reduced to 168 h, followed by a solid “plateau” for the three profiles. Additionally, the results reveal that the kinetic model that best describes the release profile of β-C from the inclusion complex is the Higuchi model (Table 2). At the same time, the transport is controlled by a combination of polymer relaxation and Fickian diffusion mechanisms, as was indicated by the Korsmeyer–Peppas model [33,34,35]. Diffusion can arise through a polymeric matrix showing the characteristic release profile, which is described by Fick’s first law, and the parameters, including temperature, concentration gradient, pH, the distance the particles must travel, polymer type, the amount of surface area available, and the molecular weight of the active compound, that may change the diffusion rate [36,37,38].

### 2.5. Sorption Studies

#### 2.5.1. Adsorption–Desorption Isotherms

**Figure 3:**
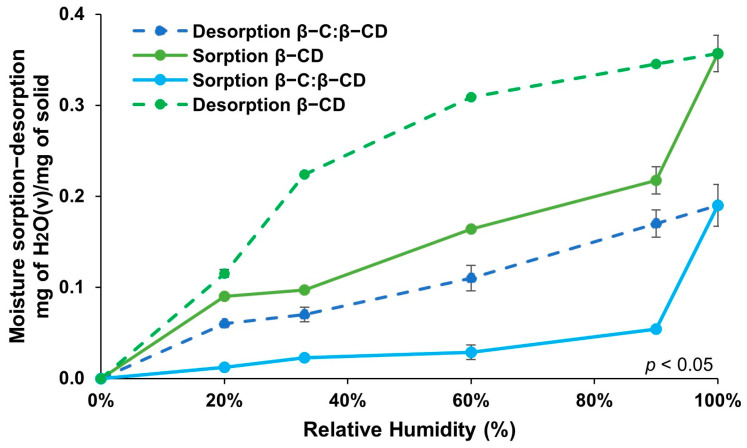
Moisture sorption and desorption isotherms for β-CD and β-C/β-CD inclusion complexes.

The assay of the adsorption isotherms provides data on the interactivity between the adsorbate and the adsorbent when the adsorption process reaches equilibrium and permits the establishment of the adsorption capacity of the substance, which is a key parameter for the system assessment [39]. The moisture sorption and desorption isotherms of the β-CD free and β-C/β-CD inclusion complex were evaluated at 8 °C (Figure 3). β-CD manifested an unceasing uptake from 33% to 90% RH with 0.1–0.2 g of water absorbed per gram at 90%. This water uptake of β-CD correlates with <20% weight change at 90% RH. These values are in agreement with the moisture content (14–15%) of β-CD at normal conditions [40]. A further 10% increase in RH (100%) resulted in a substantial increase in moisture. When the adsorption isotherm of the β-C/β-CD complex was examined, it was possible to notice a type II isotherm characterized by not reaching a saturation state. Nevertheless, the degree of adsorption tends to infinite at a partial pressure close to unity.

It is remarkable that the β-CD free and β-C/β-CD inclusion complex exhibit different adsorption–desorption profiles, designating that the occupancy of β-C in the complex influences the moisture uptake. Reasonable hysteresis was shown on both the β-CD free and encapsulated β-C. At all RHs, the free β-CD confined more water than the inclusion complex. These results may be described as the encapsulation of the β-C by the β-CD. The active compound (β-C) is in the hydrophilic sites of the β-CD and, as a result, the space of the capsules to adsorb water is reduced [41]. These outcomes could encourage the theory that β-C molecules interact with the β-CD cavity through dipole–dipole attraction (hydrogen bonds), as has been revealed with the interactions of other active compounds [10,11,42]. Moisture transfer in polymer systems is affected by the holes in the polymer matrix and of the hydrogen bonding sites through the polymeric chain, which is dependent on the attraction forces between the polymer and water molecules, because two phenomena occur consecutively: water diffusion and polymer chain relaxation [43]. According to these results, the β-C/β-CD complex is indeed a viable material for the release of active compounds like β-C due to the high adsorption of β-CD free from environments containing concentrations of water.

#### 2.5.2. Sorption Kinetics

**Figure 4:**
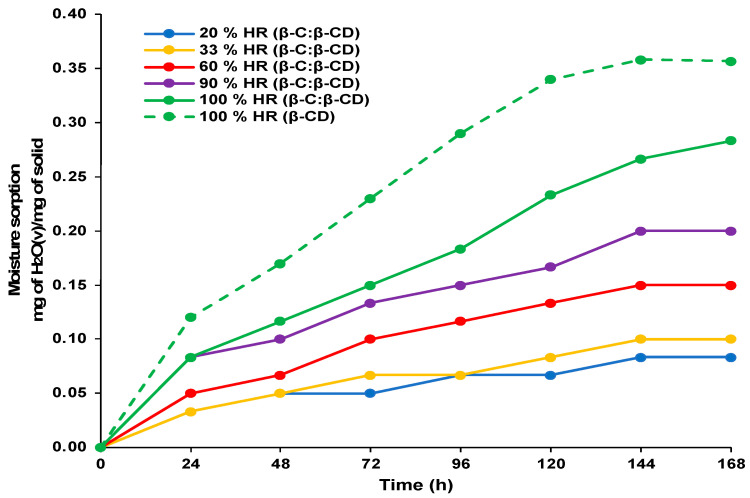
Sorption kinetic models for β-CD and β-C/β-CD inclusion complex at different relative humidities and 8 °C.

The adsorption kinetic analysis provides valuable information about the adsorption efficiency of adsorbent material and a better understanding of the adsorption mechanism [44,45]. Figure 4 represents the effect of contact time on the sorption capacities of the β-CD and β-C/β-CD inclusion complexes. The sorption capacity increased highly during 144 h of contact, and then the rate increased gradually until the equilibrium was reached. The kinetic analysis of the adsorption showed that the process reached equilibrium after 168 h for the β-CD complex and β-C/β-CD inclusion complex.

**Figure 5:**
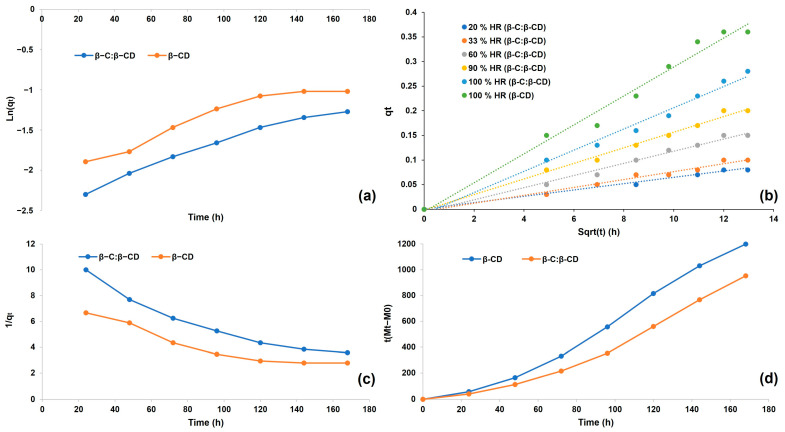
Model adjusted to (**a**) pseudo-first order, (**b**) pseudo-second order, (**c**) Peleg’s model, and (**d**) intraparticle diffusion kinetics for the β-C/β-CD inclusion complex and β-CD free.

The empirical data from the sorption kinetic curve were assessed according to the pseudo-first order (Figure 5a), pseudo-second order (Figure 5b), Peleg’s (Figure 5c), and intraparticle diffusion kinetics models (Figure 5d). The suitability of the equation was assessed based on the coefficient of determination (R^2^) (Table 3). In this present study, high R^2^ values (R^2^ > 0.99) were accomplished for the moisture sorption data, which were well fitted by Peleg’s model, suggesting the model’s accuracy in foreseeing the moisture sorption capacity. Peleg’s model was devised to describe the rehydration process. Despite being an empirical model, its constants carry physical significance. The Peleg’s constants K_1_ and K_2_, obtained from the linear fit, are presented in Table 2. A constant K_1_ provides insight into the water absorption rate, particularly in the initial phase of the process under consideration. A constant K_2_ enables the prediction of the maximum water absorption capacity. This model is extensively valuable for forecasting the rehydration progress of dried biological materials [46,47,48]. The ability of Peleg’s model to foresee the moisture equilibrium value as time approaches infinity is crucial for estimating a broad range of values using data gathered over a relatively short period [49].

The intraparticle diffusion model is used to evaluate liquid/solid adsorption kinetics (in other words, the diffusion of the adsorbate until it penetrates the adsorbent), and it describes the processes controlled by diffusion. It was shown that intraparticle diffusion is involved in the adsorption process due to a linear relationship (Figure 5d), indicating that intraparticle diffusion is the only control step. When the graphs do not pass through the origin, this indicates some degree of boundary layer uncontrol, further showing that intraparticle diffusion is not the only rate-controlling step [50,51].

### 2.6. Fourier Transform Infrared Spectroscopy (FT-IR)

**Figure 6:**
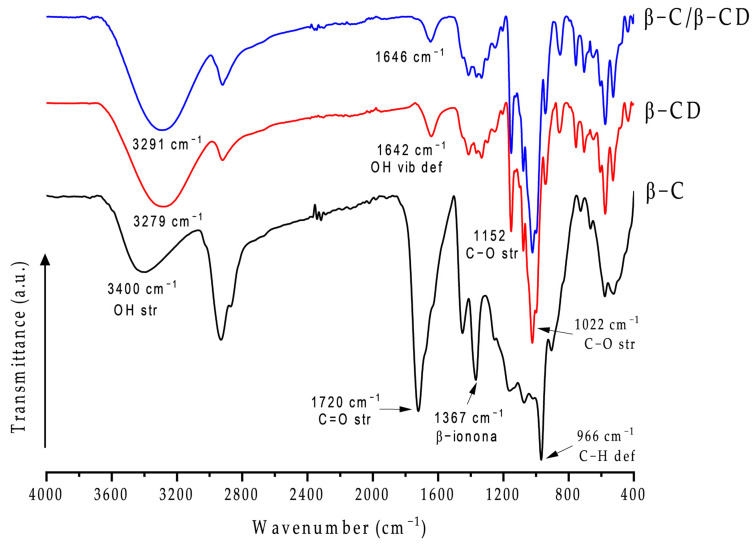
Fourier transform infrared spectroscopy (FT-IR) for β-CC, β-CCD, and β-CC/β-CCD inclusion complex.

The chemical absorption spectra of pure β-C, pure β-CD, and β-C/β-CD inclusion complexes were reported to evaluate the host–guest interaction between β-C and β-CD (Figure 6). The spectra were analyzed to investigate the interaction of β-C with β-CD based on the characteristics of the IR peaks of each component and the changes due to encapsulation (shifting, decreasing, or disappearing) [52]. Pure β-C showed a sharp peak at 966 cm^−1^ (trans-conjugated alkene C–H out-of-plane deformation mode), 1367 cm^−1^ (C–H bending), 1720 cm^−1^ (C=O bending), and 2930 cm^−1^ (C–H asymmetry stretching) [53,54]. In addition, the FT-IR spectra of β-CCD showed a clear peak at 1022 cm^−1^ (C–O–C bending), 1152 cm^−1^ (C–O–C stretching), 1642 cm^−1^ (H–O–H bending vibration), 2920 cm^−1^ (C–H stretching), and 3100–3400 cm^−1^ (O–H stretching) [55,56,57]. The inclusion complex showed almost the same peaks as β-CD, which suggests an interaction without forming a covalent bond [53]. However, some signals shifted to higher wavenumbers, such as 2920 to 2930 cm^−1^ and 3279 to 3291 cm^−1^. Likewise, the characteristic peaks of β-C, including 966, 1367, 1720, and 2930 cm^−1^, disappeared or smoothed in the spectra of the inclusion complex. This can be interpreted as hydrophobic interaction between β-C and the β-CD cavity, where hydrogen bonds were established between the hydroxyl groups of lipid with β-CD, indicating that β-C was encapsulated in the β-CD cavity, and the vibration of β-C was restricted due to the bands being shifted to lower frequencies in the spectra of the inclusion complex via a particular interaction, such as hydrogen bonds, hydrophobic interactions, or van der Waals forces [53,58,59]. All these results contribute substantial evidence of the formation of an inclusion complex.

### 2.7. Raman

**Figure 7:**
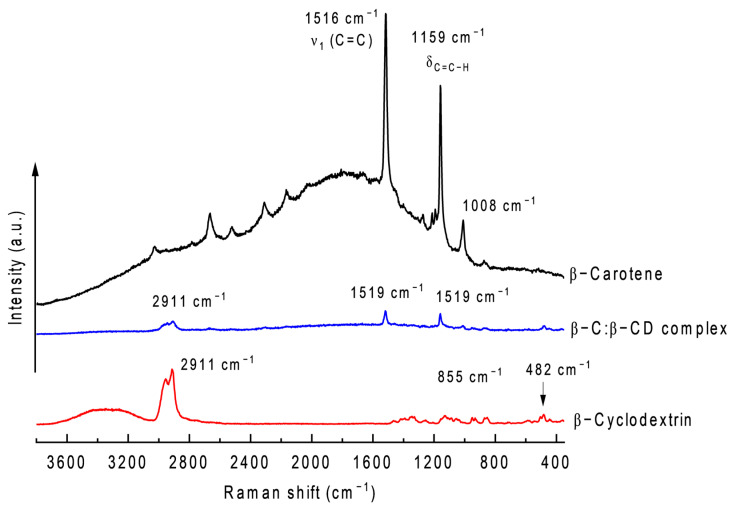
Raman spectra of β-C, β-CD, and β-C/β-CD inclusion complex.

The Raman vibrational spectra of the pure β-CC, pure β-CCD, and β-CC/β-CCD inclusion complex are shown in Figure 7. The Raman spectra of β-CC showed intense bands at 1516 cm^−1^, 1159 cm^−1^, and 1008 cm^−1^, which are attributable to the C=C stretching mode (ν_1_), C–C in-plane single bond stretching mode (ν_2_), and C–H bending mode, respectively [53,60,61]. β-CCD showed peaks at 482 cm^−1^, 855 cm^−1^, and 2911 cm^−1^, attributable to the vibration of the C=O and C–C bonds and glucopyranosyl ring structure, the wagging-type vibration modes of the hydroxyl bonds of sugar rings, and the aliphatic C–H, CH_2_ stretching vibration mode (symmetric and asymmetric bonds) [57]. The spectra of the β-CC:β-CCD inclusion complex exhibit a slight blue shift for ν_1_ (Δν = −3 cm^−1^). Although this is a very small change, it has been documented that it is associated with a possible conformational change caused by the confinement process of β-Ccarotene [57,60].

### 2.8. Differential Scanning Calorimetry (DSC)

**Figure 8:**
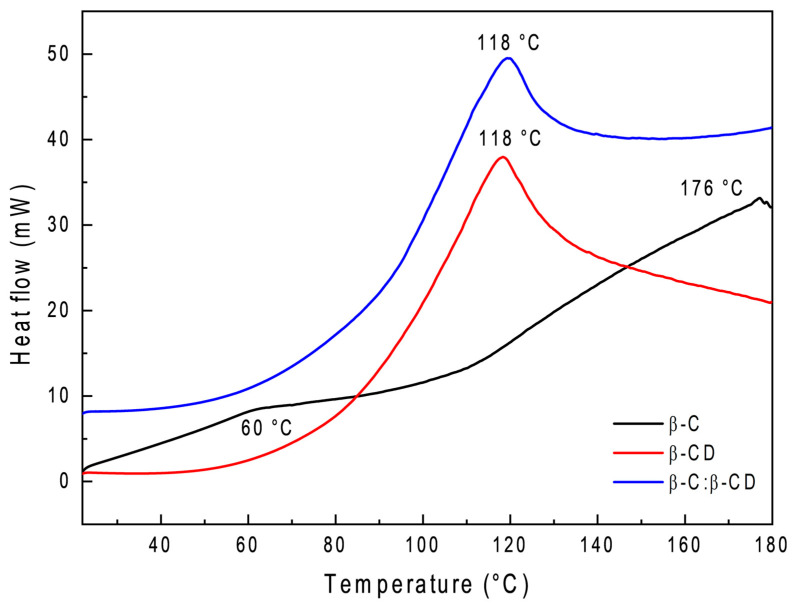
DSC thermogram of β-C, β-CD, and β-C/β-CD inclusion complex.

DSC is a thermal analysis method employed to track various alterations in a sample due to temperature variations. It is particularly useful for examining crystalline substances’ melting and recrystallization behaviors [62]. The DSC curves of the β-C, β-CD, and β-C:β-CD inclusion complexes are shown in Figure 8. Their thermal stability was evaluated based on the variances observed in phase transitions throughout the heating process. The findings offer a valuable understanding of the solid-state interactions between the β-C and β-CD. As shown in Figure 8, β-C showed a decomposition peak at 60 °C, and another peak appeared at 176 °C corresponding to the release of volatile compounds. An endothermic peak for β-CD at 118 °C appears in its first stage, attributed to the escape process of water molecules in the cavity due to evaporation. In the case of β-C/β-CD, changes were noted in the thermograms after encapsulation. No typical endothermic peaks of β-C were seen in the complex, confirming the suspicion that carotene has been defended within the hydrophobic cavity. No less importantly, the decrease in the height of the endothermic peaks and the decline in the area corresponding to the H delta may represent the loss of water molecules in the β-CD cavity due to the encapsulation of β-C and the rearrangement of the crystalline structure [63].

### 2.9. Thermogravimetric Analysis (TGA-DTG)

**Figure 9:**
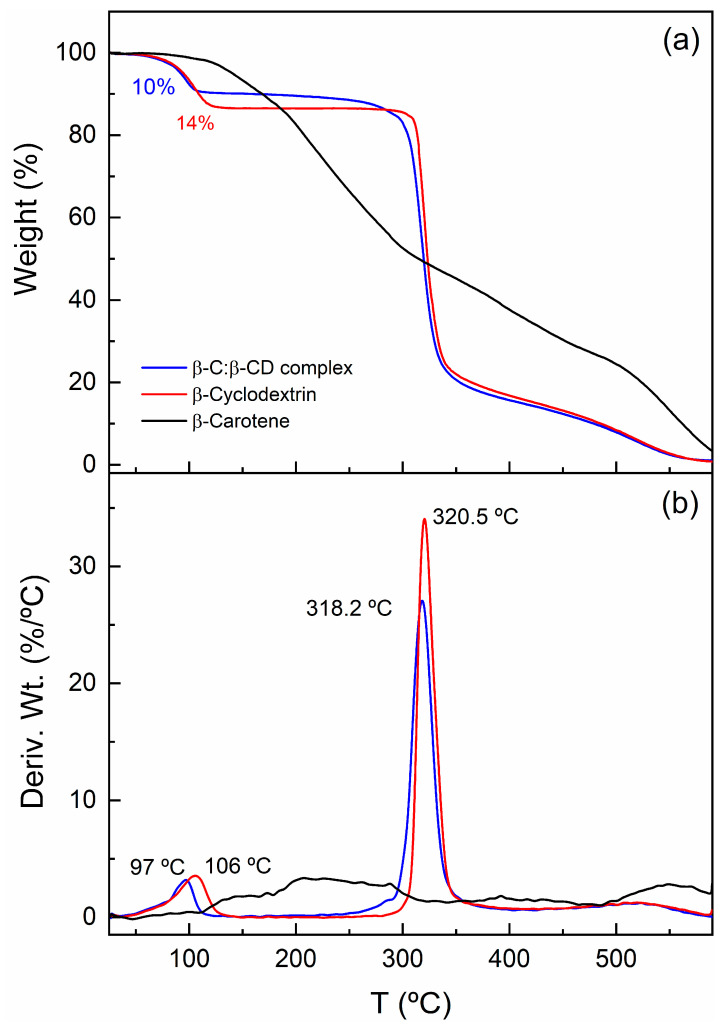
Thermogravimetric (**a**) and differential thermogravimetric (**b**) curves of β-C, β-CD, and β-C/β-CD inclusion complex.

Thermogravimetric (TG) and differential thermogravimetric (DTG) curves for the β-C, β-CD, and β-C/β-CD inclusion complex are displayed in Figure 9. The thermogram of β-C exhibits a weight loss starting at 117 °C and proceeds through a series of indistinguishable consecutive steps, which are associated with the decomposition of the compound. On the other hand, cyclodextrin exhibits two notable thermal events. The first occurs at 106 °C, which is associated with water loss (14 wt.%). The second thermal step peaks at 320 °C, with a weight loss of approximately 66%; this event is associated with the decomposition of the polymer. In the case of the inclusion complex, the thermal behavior displays patterns similar to those of the oligosaccharide, although some differences can be identified. The β-C/β-CD system exhibits a water loss due to evaporation equivalent to 10 wt.% at a maximum value of 96 °C. Furthermore, it is observed that the temperature at which water molecules are lost in the β-CD structure is higher (Δ*T* = 9 °C) than that observed in the β-C/β-CD complex. In this sense, it is known that water can bond in polymeric networks by different routes: bulk water, structured water (hydrophobic interactions), hydrogen bonding, and polarized water around charged ionic groups [64]. In the case of cyclodextrin, the hydrophobic cavity present in its structure could attract water molecules of the “structured water” type, while, on the surface, it would mostly have “bulk water” interactions. It is known that water evaporation below 100 °C is due to free or surface water, while water loss above 100 °C is associated with structured water molecules bound to the molecule by secondary interactions such as van der Waals and hydrogen bonds [65]. Then, it can be hypothesized that the decrease in water weight loss in the inclusion complex with respect to β-CD may be due to the confinement of β-carotene in its cavity, causing a reduction in its capacity to capture water molecules inside.

### 2.10. Scanning Electron Microscopy (SEM)

**Figure 10:**
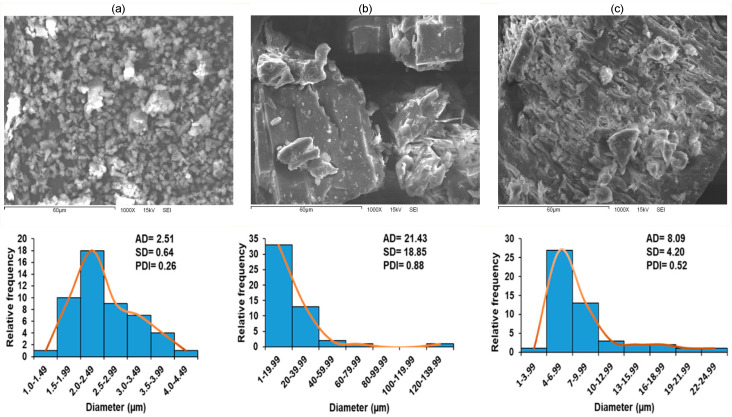
SEM images and particle size of (**a**) β-C, (**b**) β-CD, and (**c**) β-C/β-CD. AD = average diameter; SD = standard deviation; and PDI = polydispersity index.

The microscopic surface morphologies of the particles of the β-C/β-CD inclusion complex were qualitatively observed to determine the complexation phenomenon’s structural aspects. Figure 10 illustrates the topography of the scanning electron micrographs of the pure β-C, pure β-CD, and β-C:β-CD inclusion complex. β-C (Figure 10a) was observed to have an irregular spherical shape in the form of tiny aggregates, while the β-CD (Figure 10b) particles had an irregular crystal form with different and flake-dense structures [57]. The case of the β-C/β-CD inclusion complex (Figure 10c) showed a remarkable variation compared to β-C and β-CD in shapes and sizes, resulting in a combination of the parent compounds with the appearance of small irregular-shaped clumps in the surface.

Figure 10 also contains the histograms of the particle size of the samples. In the first histogram, representing commercial (a) β-C, the particle size distribution is narrow, with an average diameter (AD) of 2.51 µm, a standard deviation (SD) of 0.64 µm, and a polydispersity index (PDI) of 0.26. These results indicate a monodisperse sample characterized by a uniform particle size distribution, as evidenced by the low SD and PDI. The small size and narrow distribution suggest that β-C, in its commercial form, is relatively homogeneous and does not form large aggregates, making it suitable for direct use in applications requiring small and uniform particles.

In the second histogram, for (b) β-cyclodextrin, the distribution is much broader, with an AD of 21.43 µm, an SD of 18.85 µm, and a high PDI of 0.88. This wide size distribution reflects significant variability in particle sizes, likely due to the inherent structural properties of β-cyclodextrin, which tends to form larger and irregular agglomerates. The high SD and PDI confirm that this is a polydisperse system, suggesting that β-cyclodextrin, in its raw form, may not be ideal for applications requiring fine particle size control. The broad range of sizes, from very small to very large, can contribute to instability in formulations and may hinder performance in specific applications.

In the third histogram, corresponding to (c) the β-C/β-CD inclusion complex, the particle size distribution lies between the previous two, with an AD of 8.09 µm, an SD of 4.20 µm, and a PDI of 0.52. This distribution indicates a transition towards a more monodisperse sample compared to pure β-CD, with a lower PDI suggesting a narrower size range. However, it is still not as uniform as commercial β-C. The formation of the inclusion complex results in a reduced particle size and improved uniformity, as the interaction between β-C and β-CD likely stabilizes the β-C and prevents the formation of larger agglomerates seen in pure β-CD.

### 2.11. X-Ray Diffraction (XRD)

**Figure 11:**
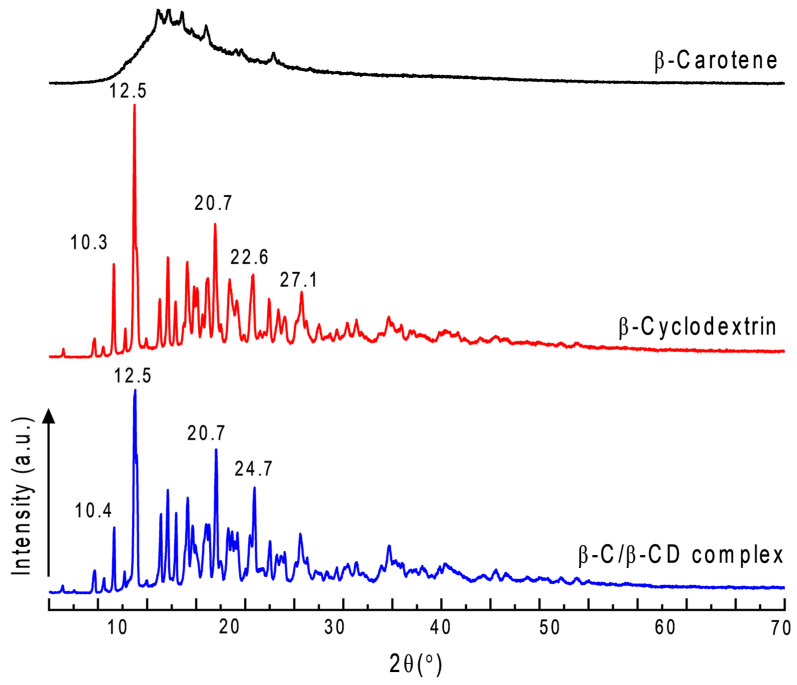
Diffractogram of β-C, β-CD, and β-C/β-CD inclusion complex.

The inclusion complex of β-C with β-CD was investigated further by using XRD. Figure 11 shows the X-ray diffraction patterns of pure β-C, pure β-CD, and the β-C/β-CD inclusion complex. Pure β-C exhibited sharp diffraction peaks (2θ) of 10–30°, indicating the crystalline state of the molecule [53,54,66]. The β-CD showed many characteristic peaks in 12.5, 22.6, and 27.1, with similar results found in the literature [53,58]. However, the inclusion complex of β-C with β-CD appears to have new diffraction peaks throughout the pattern, indicating the preservation of the crystalline form for the inclusion complex. The diffractogram demonstrated a crystalline structure, which may be issued from β-C entrapped in the cavity interior of β-CD. Qualitatively, the partial diffraction peaks approximate to β-CD revealed that β-C/β-CD have a higher proportion of β-CD participating in the complexation and determined the interaction. The above results supported that β-C and β-CD formed inclusion complexes in the solid phase with unique characteristics.

### 2.12. Zeta Potential (ζ)

As a necessary standard to evaluate stability, indicating the surface charge, the zeta potential (ZP) refers to the electrostatic repulsion in colloidal dispersions. The ZP of the inclusion complex was found to be −28 ± 1.8 mV; the negative charge is due to the free hydroxyl groups on the surface. Shende et al. [67] reported that the zeta potential of β-CD is 23.9 ± 0.71 mV; they also observed an increase in zeta potential (up to −31.06 ± 1.31 mV) with an increasing encapsulated drug. The charge provided by β-C increased the mass of the electron cloud, leading to greater stability of the particles of the β-C/β-CD inclusion complex.

### 2.13. Antioxidant Activity

The antioxidant activity of the β-C/β-CD complex was assessed using ABTS, DPPH, and FRAP assays (Table 4) to evaluate the impact of β-CD complexation on its antiradical activity and electron transfer potential. The results show a dose–response relationship. The maximum radical inhibition obtained by the complex in the highest concentration was 34.09% in ABTS and 21.73% in DPPH. These results were lower than the inhibition showed by ascorbic acid (74.33% ABTS and 61.93). In the FRAP, the complex showed 8.85 M TE/g, similar to ascorbic acid (8.34 μM TE/g). Notably, the inhibition percentages of β-C in the complex were comparable to those of pure β-C across all the assays, indicating that the inclusion complex preserves the biological activity of β-C. Similarly, the FRAP results confirmed that the electron transfer potential of β-C remains intact within the complex.

### 2.14. Protective Effect on Human Erythrocytes

It is well established that CDs can enhance the bioavailability of compounds encapsulated within their cavity [68,69,70,71,72]. Once bioactive compounds are absorbed through the intestine, they enter the systemic circulation. However, limited research has focused on their erythroprotective effects, as most studies primarily examine their impact on specific organs or target sites of action.

For this reason, interest arose in evaluating the protective effect of the β-C/β-CD inclusion complex (Table 5). The amount of β-C studied was the same as that in the β-C/β-CD complex. Table 5 shows that the greater protection of erythrocytes was observed with the β-C/β-CD inclusion complex with the highest concentration (64.09% of hemolysis inhibition) than with pure β-C (51.33% of hemolysis inhibition), which means, in this case, the effect of encapsulation in β-CD increased its erythroprotective activity, inhibiting the damage caused by the generation of radicals, principally peroxyl radicals, through APPH.

## 3. Discussion

The precipitation method employed to obtain the β-C/β-CD complexes proved to be a suitable method to complex β-C into β-CD with a high yield and EE%. The efficiencies of carotenoids, such as β-C, are challenging to determine due to the molecular hydrophobicity, their capability to fit into the complex, and their stability during the integration process in the cavity [73]. However, since the bond is established between the hydrophobic section, hydrophobic molecules like this are most favorable concerning an increment in their water solubility [74]. This was also corroborated by the FT-IR and Raman, which demonstrated the possible interactions among β-C with β-CD by hydrogen bonds, hydrophobic interactions, or van der Waals forces. Similar results can be found, as reported previously [61]. The variances in the vibrational intensities of the predominant bonds are due to the cramped vibration of the aromatic ring structure of β-C moiety in the β-CD cavity [55,57]. Additionally, XRD supported that β-C and β-CD formed inclusion complexes in the solid phase with unique characteristics. The ZP measurements indicated a negative charge (−23 to −32 mV), suggesting improved surface charge stability due to the formation of hydrogen bonds between β-CD and the hydroxyl groups of β-C. These results define the inclusion complex as having enhanced stability and solubility, driven by the synergistic interaction between the components. The ZP values were sufficiently high to confirm the stability of the dispersions, indicating a low risk of aggregation over time.

Numerous studies have reported that the encapsulation of bioactive compounds within the β-CD cavity often results in the disappearance or reduced intensity of their characteristic thermograms, as observed in our findings [57,58,59,75,76]. These changes confirm the occurrence of complexation and highlight the stability of the β-C/β-CD inclusion complex. Replacing water molecules near the inner cavity of β-CD with β-C significantly influences van der Waals interactions and hydrogen bonding, forming a thermally stable complex. These results support the theory that β-CD enhances the thermal stability of β-C through encapsulation, protecting against oxidation and decomposition [77].

Several factors, including the type and concentration of the carrier and the temperature conditions during the drying process, can influence powder functionality. Consequently, it is important to determine moisture content [78,79]. Previous studies align with our findings, reporting a moisture content of around 13% for β-CD alone, which suggests that β-CD can retain more water externally when it does not encapsulate another compound [42]. This behavior could be advantageous for retaining moisture in high-water-content foods, such as minimally processed fruits. For example, fresh-cut fruits lose water rapidly, leading to oxidative reactions and microbial growth, which cause spoilage and significantly reduce their shelf life [42]. The β-C/β-CD inclusion complex could be utilized as a time-release system to extend the shelf life of these fruits during storage, reducing waste and preserving quality.

Studies were conducted with different relative humidities (RH 0–100%) to determine the release behavior under varying conditions. A positive correlation was observed in the release of β-C when the RH increases. This indicates that the more water molecules that bind to the outside of the inclusion complex, the more they will release the β-C. Release studies were performed at 8, 25, and 37 °C. However, β-C released higher at 37 °C, which could be attributed to the upper temperature that increments the molecular momentum gradient in the solution medium. Temperature can influence concentration gradients through its impact on molecular movement and diffusion. Diffusion is the movement of particles from an area of higher concentration, which, in this case, is the inclusion complex, to an area of lower concentration, which is the aqueous medium [45,80,81].

The results suggest that the release kinetics are controlled solely by diffusion through the polymer surface, as found in Horablaga et al. [82]. The mathematical modeling of the bioactive compound release aims to prognosticate the release rates and diffusion nature from transport systems. These data support the optimization of the release kinetics and the forecast of the physical mechanisms involved in delivery, which is simplified by comparing the experimental results with mathematical models. The Korsmeyer–Peppas model was the best to describe the transport of β-C. This model is generally used to analyze the release of polymeric dosage forms in pharmaceuticals when the delivery mechanism is not fully known or when more than one type of release phenomenon could be involved [37], which is the case of our samples. However, the kinetic model that best describes the release profile of β-C from the inclusion complex was the Higuchi model, indicating the release of β-C from β-CD when the drug load exceeds its solubility limit.

The morphology of small irregular-shaped clumps in the surface of the β-C/β-CD inclusion complex is due to the change in the characteristic structure of cyclodextrins, suggesting the successful formation of an inclusion complex. Multiple morphologies of various inclusion complexes can be observed, which may be based on the admitting ability of the β-CD cavity on various guest molecules to encapsulate. This behavior revealed the interaction between β-C and β-CD and the accomplishment of encapsulation [58]. On the other hand, the histograms illustrate the effectiveness of β-CD in encapsulating β-C to form smaller, more stable complexes, while highlighting the differences in particle size distribution across the three systems. The encapsulation process significantly improves the uniformity and size control of the material, making it potentially more suitable for applications requiring enhanced bioavailability and stability compared to raw cyclodextrin. The differences in monodispersity and polydispersity among the samples underline the importance of particle size distribution in determining the suitability of materials for specific applications.

The β-C/β-CD inclusion complexes maintained the stability of β-C in terms of its antioxidant capacity, since no significant difference was observed with pure β-C. This demonstrates that β-CD stabilizes the bioactivity of compounds like this, and these results follow some studies [83,84,85]. It can also be seen that the amount of DPPH is less than ABTS in all the samples, probably due to ABTS’s affinity for hydrophilic and hydrophobic compounds covering a wider range of compounds that the DPPH does not consider [86,87]. Generally, these assays assess the antioxidant mechanism based on electron transfer; however, they lack specificity toward particular types of antioxidants. FRAP is an exclusive electron transfer technique that measures the reducing power of iron. The FRAP values were low; therefore, it cannot be completely inferred that the mechanism of action is entirely by electron transfer. Further studies will be required to pinpoint the mechanism.

This study induced radicals, mainly peroxyls, in erythrocytes from AAPH to test the effectiveness of the β-C/β-CD inclusion complexes. It is important to note that radicals are potent oxidants and key contributors to chronic degenerative diseases. By protecting erythrocytes from these radicals, the development of such conditions could potentially be mitigated. The β-C/β-CD inclusion complex demonstrated an erythroprotective effect. Some studies carried out by our research group indicate that the specificity of bioactive compounds in erythrocytes is influenced by the surface antigens (A, B, AB, and O), as well as the “+” or “−” Rhesus factor [88]. In our case, only the blood type A Rh + could be studied, and it is probable that, if we study the different blood groups in the future, we could find different percentages of hemolysis inhibition. With this information, these compounds could be applied with greater specificity depending on the blood groups, making them more effective. Therefore, more studies are needed. To our knowledge, the antihemolytic activity of β-carotene encapsulated in β-CD or similar systems has been scarcely reported in the literature. This limited availability of data constrains direct comparisons with other delivery systems. To provide context for our findings, we compared the hemolysis inhibition values of the β-C:β-CD complex with those of unencapsulated antioxidants. Systems based on natural antioxidants such as quercetin, fucoxanthin, β-carotene, gallic acid, and ascorbic acid have demonstrated antihemolytic activity against AAPH-induced oxidative hemolysis in various ABO RhD+ve blood types, with reported inhibition values ranging from 80% to 100% [88]. Furthermore, a separate study revealed that quercetin reduced AAPH-induced hemolysis in human red blood cells by 88% compared to the vehicle, while other compounds such as ascorbic acid, α-tocopherol, and α-tocotrienol failed to achieve significant reductions in hemolysis [89]. Although the antihemolytic activity observed for the β-C:β-CD complex in this study was lower than those reported for some unencapsulated antioxidants, its additional benefits, such as enhanced β-carotene solubility, stability, and bioavailability, make it a promising system for mitigating oxidative damage. These properties could broaden its potential applications in the food and pharmaceutical industries, particularly in formulations targeting oxidative stress-related conditions.

In practical applications such as food preservation and pharmaceuticals, the β-C/β-CD inclusion complex offers several advantages over existing delivery mechanisms. First, β-CD enhances the thermal and oxidative stability of β-carotene (β-C), protecting it from the degradation caused by light, oxygen, and heat. This is particularly beneficial in food preservation, where β-C/β-CD can extend the shelf life of products by reducing oxidation and maintaining antioxidant activity. Additionally, β-CD’s ability to improve the solubility and bioavailability of β-C enables its incorporation into aqueous systems, broadening its application in liquid and semi-solid formulations. Compared to other delivery systems, such as liposomes or emulsions, β-CD does not require complex preparation methods, reducing production costs and scalability challenges. Furthermore, β-CD is a solid material that is easy to handle and can be directly incorporated into powders or encapsulated systems for pharmaceuticals, facilitating storage and transport. Its biocompatibility and non-toxicity also make β-CD a safer alternative, aligning with regulatory requirements for food and pharmaceutical applications. These advantages position β-C/β-CD as an effective and versatile industry delivery mechanism.

## 4. Materials and Methods

### 4.1. Materials

The β-Carotene (β-C), β-Cyclodextrin (β-CD) with a molecular weight of 1134.98 g/mol, 2,2′-Azobis(2-amidinopropane) dihydrochloride (AAPH), 2,2′-azinobis(3-ethylbenzothiazoline)-6-sulfonic (ABTS), 2,2-diphenyl-1-picrilhydrazil (DPPH), 2,4,6-tripyridyl-s-triazine (TPTZ), hydrochloric acid (HCl), Triton X-100, and phosphate buffer saline (PBS) were purchased from Sigma-Aldrich (St. Louise, MO, USA). All the other chemicals and materials were of analytical grade.

### 4.2. Preparation of β-C/β-CD Inclusion Complex

The precipitation method of Perez-Perez et al. [82] was employed with some modifications. Briefly, β-CD was dissolved in ethanol–water (1:2) at 55 °C, while β-C was dissolved in ethanol (10% *w*/*v*). The β-C solution was added slowly to the β-CD solution to obtain a ratio (β-C/β-CD) of 40:60 (% *w*/*w*). After stirring for 4 h at 25 °C in darkness, the sample was maintained at 4 °C for 12 h. The solution was centrifuged at 2500× *g* for 10 min. Two washes were then performed with the same solvent and centrifuged again. The precipitate formed was dried at 50 °C for 24 h. The recovered inclusion complex (Yield%) was calculated using Equation (1).

(1) $$Yield\left(\%\right)=\frac{total\;weight\;of\;inclusion\;complexes}{initial\;weight\;of\;\beta -CC+initial\;weight\;of\;\beta -CCD}\times 100$$

### 4.3. Entrapment Efficiency (EE%) and Loading Efficiency (LE%)

EE% and LE% were determined using the methodology indicated by Huang et al. [90]. The β-C/β-CD inclusion complex (1 g) was dissociated using 5 mL of ethanol and vortexed for 3 min. Subsequently, the β-C/β-CD inclusion complex was centrifuged (4000 rpm, 5 min), and the fraction of β-C in the organic solvent was quantified based on a calibration curve (0–0.1 mg/mL). Each experiment was carried out in triplicate. The determinations were carried out using UV–visible absorption spectrophotometry (Multiskan Go, Thermo Scientific, Waltham, MA, USA) at 450 nm with ethanol as the blank with the following Equations (2) and (3):

(2) $$EE\left(\%\right)=\frac{weight\;of\;encapsulated\;\beta -CC}{initial\;weight\;of\;\beta -CC}\times 100$$

(3) $$LE\left(\%\right)=\frac{weight\;of\;encapsulated\;\beta -CC}{total\;weight\;of\;inclusion\;complexes}\times 100$$

### 4.4. UV–Visible

Using UV–visible spectroscopy (Multiskan Go, Thermo Scientific, Waltham, MA, USA), the spectral alterations that occurred during complexation were identified. Free β-carotene was eliminated by filtering the inclusion complexes (1 mg) after they were dissolved in ethanol (10 mL) for the co-precipitated complex. Pure B-carotene and B-cyclodextrin were prepared in ethanol at the same concentration that was in the complex. The 300–500 nm range is where the spectra were recorded.

### 4.5. Moisture Content

Samples (1 g) in triplicate were placed in an oven, BOEKEL model 132,000 (Feasterville, PA, USA), at 50 °C for 24 h and subsequently in a desiccator with a vacuum for 3 h to stabilize the temperature. Finally, the sample was weighed on a precision balance OHAUS model AX423/E (USA) [91].

### 4.6. Release Studios

The release profile of β-C from the β-C/β-CD inclusion complex was studied at three different temperatures (8, 25, and 37 °C) for 240 h. β-C was determined by UV–visible as described in Section 4.3. The cumulative release rate (%) of β-C was plotted as a function of time as Equation (4):

(4) $$(\%)=\sum_{0}^{t}\left(M_{0}/Mt\right)$$

where M_0_ and M_t_ are the initial amount of β-C encapsulated and the cumulative amount of released β-C in the aqueous media, respectively. Mathematical studies analyzed the release profile data following the zero (Equation (5)) and first order (Equation (6)), Higuchi (Equation (7)), and Korsmeyer–Peppas (Equation (8)) models to evaluate which release mechanism fitted better [92,93].

(5) $$Q_{t}=Q_{0}+K_{0}t$$

(6) $$logC=logC_{0}+\frac{Kt}{2.303}$$

(7) $$Q_{t}=k_{H}t^{1/2}$$

(8) $$\frac{M_{t}}{M_{\infty }}=K\cdot t^{n}$$

where Q_t_ = the quantity of released β-C at a specific time; Q_0_ = the initial amount of β-C in the solution; C = concentration; C_0_ = initial concentration; t = time; K, K_0_, k_H_ = rate constant; M_t_/M_∞_ = the total amount of β-C released at time t; and n = a particular diffusion mechanism.

### 4.7. Adsorption–Desorption Isotherms

The kinetics of the β-C/β-CD inclusion complexes were determined when they adsorbed moisture and hydrated (the adsorption isotherm). On the other hand, the dehydration process and how the complexes lose water (the desorption isotherm) were also determined. Considering the future application of the β-C/β-CD inclusion complexes in the storage of fresh-cut fruits, we selected a temperature of 8 °C, the temperature at which most of the products are exhibited on ice to keep them fresh. For the adsorption isotherm, the samples (25 mg) were subjected in closed containers to a different RH (20, 33, 60, 90, and 100%) using saturated salt solutions (dihedrite, MgCl_2_, NaBr, BaCl_2_, and H_2_O, respectively) for 21 days at 8 °C. For the desorption, it was the same procedure using the same series of RHs but in reverse order, according to Perez-Perez et al [87]. The data are reported as the percentage of adsorbed or desorbed water. The determinations were carried out in triplicate.

### 4.8. Sorption Kinetics

The adsorption data for the water uptake versus contact time at different moistures were obtained to evaluate the maximum amount of entrapment until constant values. Furthermore, the mechanism of sorption was studied. Four kinetics models have been proposed for the sorption processes to find the best-fitted model for the data obtained [51,93]: the pseudo-first order (Equation (9)), pseudo-second order (Equation (10)), Peleg’s (Equation (11)), and intraparticle diffusion models (Equation (12)).

(9) $$\log\left(q_{e}-q_{t}\right)=\log q_{e}-\frac{Kt}{2.303}$$

(10) $$\frac{t}{q_{t}}=\frac{1}{Kq_{e}^{2}}+\frac{t}{q_{e}}$$

(11) $$M_{t}=M_{0}+\left(\frac{t}{K_{1}K_{2}t}\right)$$

(12) $$q_{t}=Kt^{1/2}+C$$

where q_e_ = the quantity sorbed athe t equilibrium, q_t_ = the quantity sorbed at a specific time, K= rate constant, t = time, M_t_ = moisture content at a specific time, M_0_ = maximum water absorption capacity (equilibrium moisture content), K_1_, K_2_= Peleg’s kinetic parameters, and C = Weber–Morris diffusion constant.

### 4.9. Fourier Transform Infrared Spectroscopy (FT-IR)

To determine the chemical–structural interactions of β-C with β-CD, we used FT-IR. The infrared spectrum of the samples was obtained in a Perkin Elmer FTIR Frontier model (Waltham, MA, USA) in combination with an accessory to analyze the attenuated total reflectance, with a wave number resolution of 0.10 cm^−1^ in the range of 400–4000 cm^−1^ at 25 °C. A minimum of 32 scans with a resolution of 4 cm^−1^ were averaged over the above ranges [87]. The comparative FT-IR spectral data of the β-C/β-CD inclusion complexes, pure β-CD, and pure β-C were discussed.

### 4.10. Raman Spectroscopy

The analysis was carried out on a Witec Alpha 300RA brand equipment (WITec, Ulm, Germany), at 5 mV, for 5 s, with three repetitions. The laser was 532 nm long, and the 50× objective was used.

### 4.11. Differential Scanning Calorimetry (DSC)

The change in the physical properties of the β-C/β-CD inclusion complex was determined by comparing against pure β-CD and pure β-C according to Robles-García et al. [94]. The thermal behavior was studied by DSC using a Perkin Elmer model 8500 (Perkin Elmer, Shelton, CT, USA). Approximately 5 mg of the sample was placed in a stainless-steel cell, and adequate contact between the sample and the lower face of the capsule was ensured. As a reference, an empty aluminum pan was utilized. All the samples were scanned under a nitrogen atmosphere at a heating rate of 10 °C·min^−1^ from 25 to 180 °C. The melting temperature and enthalpy of the films were automatically calculated by the software provided with the equipment (Pyris version 11).

### 4.12. Thermogravimetric Analysis (TGA)

The TG and DTG profiles of the samples β-C, β-CD, and β-C/β-CD complex were obtained using TGA-DSC equipment (STA 449 F3 Jupiter; Netzsch, Selb, Germany). The samples were analyzed in an aluminum crucible in a range of RT to 600 °C by the dynamic high-resolution method at 10 °C min^−1^, under 10 mL min^−1^ of nitrogen flow.

### 4.13. Scanning Electron Microscopy (SEM) and Particle Size Distribution

The morphology of the inclusion complexes was examined using a JEOL 5410LV (SEM), operated at 15 kV. The samples were gold-sputtered before the SEM examination. Additionally, the β-C, β-CD, and β-C/β-CD complexes were evaluated through the particle size distribution analysis and average diameter using the ImageJ software (developed by the NIH, Bethesda, MD, USA; https://www.google.com/url?sa=t&source=web&rct=j&opi=89978449&url=https://imagej.net/ij/&ved=2ahUKEwjm4KnEqN-MAxVQD0QIHfJTL2kQFnoECB0QAQ&sqi=2&usg=AOvVaw00Yf37R8bXL8VTD6xpdlus, accessed on 29 December 2024). These determinations were made based on images obtained from SEM. The PDI, which provides information on the heterogeneity of particle sizes, was also calculated using the following Equation (13).

(13) $$PDI=\frac{\sigma }{X}$$

where σ is the standard deviation, and X is the average.

This index is crucial for determining whether the sample is monodisperse or polydisperse; a sample is considered monodisperse when the PDI is less than 0.3, indicating a uniform and homogeneous particle size distribution. In contrast, a sample is classified as polydisperse when the PDI exceeds 0.3 to 1.0, reflecting greater variability in particle sizes. The average size and size distribution significantly affect the particles’ physical properties, stability, and behavior in various biological systems, making it especially relevant for applications involving β-C, β-CD, or their complexes.

### 4.14. X-Ray Diffraction (XRD)

The powder-form samples were packed into the X-ray holder from the top before the analysis. The diffraction powder patterns were collected at room temperature, 40 kV, and 30 mA on a Bruker D8 Advance diffractometer (Karlsruhe, Baden-Württemberg, Germany) with a graphite monochromator and a tube anode Cu (λ = 1.54). The diffractograms were acquired in the 2θ angle range of 5–40° and from process parameters with a scanning speed of 0.04 θ/s.

### 4.15. Zeta Potential (ζ)

The samples’ zeta potential was determined using a Zetasizer Nano ZS (Malvern Instruments, Malvern, UK). Approximately 1 mg of the sample was dispersed in 1 mL of MilliQ water. During the zeta potential analysis at 25 °C, the prepared dispersions were placed in the electrophoretic cell, and the particles were moved to the electrode that had a charge opposite them after applying an electric field (15 V/cm). The measurements were performed in triplicate for at least ten determinations for each sample, and the data were expressed as the mean ± standard deviation [72].

### 4.16. Antioxidant Activity

The antioxidant activity of β-C (0.01–0.5 mg/mL), β-CD (0.01–0.5 mg/mL), and β-C/βCD (0.03–1.5 mg/mL) was determined by the methods of ABTS (2,2′-azinobis(3-ethylbenzothiazoline)-6-sulfonic) [95], DPPH (2,2-diphenyl-1-picrilhydrazil) [96], and FRAP (ferric reducing antioxidant power) [97]. The solvent (ethanol) was used as the negative control, and ascorbic acid was used as the positive control. The amount of β-C/βCD (0.03–1.5 mg/mL) was calculated to correspond to the amount of complexed β-C (0.01–0.5 mg/mL).The absorbance of the samples was made using a UV–visible microplate reader (Thermo Fisher Scientific Inc. Multiskan GO, New York, NY, USA).

#### 4.16.1. ABTS^•+^ Assay

For this assay, the radical formation (ABTS^•+^) was performed using 3.87 mg/mL of ABTS salt with 88 μL from a K_2_S_2_O_8_ solution (0.0378 g/mL). This preparation was left for 12 h at room temperature in darkness. Subsequently, ABTS^•+^ was adjusted to an optical density of 0.700 ± 0.05 at 734 nm, diluting with ethanol. The β-C and β-C/β-CD inclusion complexes (20 μL) were mixed with the ABTS^•+^ radical solution (270 μL). The absorbance was read at λ = 734 nm after incubation for 30 min. The samples were expressed as ABTS’s percentage inhibition according to Equation (14).

(14) $$\%\;of\;inhibition\;ABTS=\frac{inicial\;absorbance-final\;absorbance}{inicial\;absorbance}\times 100$$

#### 4.16.2. DPPH Assay

A DPPH stock solution (6 × 10^−5^ mol/L) was prepared with ethanol to reach an absorbance of 0.7 ± 0.02 at 515 nm. Subsequently, 20 μL of the samples were combinate with 200 μL of the DPPH solution. The combination was kept in the darkness at 25 °C for 30 min. Absorbance was obtained at λ = 515 nm, and the percentage inhibition of DPPH was calculated using Equation (15).

(15) $$\%\;of\;inhibition\;DPPH=\frac{inicial\;absorbance-final\;absorbance}{inicial\;absorbance}\times 100$$

#### 4.16.3. FRAP Assay

The FRAP assay was measured by the reducing power of the ferric ion (Fe^+3^) to ferrous ion (Fe^+2^). The FRAP solution was prepared in a 1:1:10 ratio of 10 mM of TPTZ in 40 mM of HCl, 20 mM of FeCl_3_·6H_2_O, and 0.3 M of sodium acetate buffer (at a pH of 3.6), respectively. The samples of β-C and β-C/β-CD (20 µL) were mixed with the FRAP solution (280 µL). This preparation was incubated for 60 min at room temperature. The absorbance was read at λ = 638 nm. The results were expressed as micromoles of Trolox Equivalents by a gram of sample (µM TE/g of dried sample) based on a Trolox calibration curve.

### 4.17. Protective Effect on Human Erythrocytes

In this assay, the sample’s capacity to inhibit the induced radicals formed in human erythrocytes was carried out according to the method of Hernández-Ruiz et al. [98]. Erythrocytes were obtained from healthy adult volunteers (19–45 years old) with the blood type A+ following the regulations outlined by both the Mexican (NOM-253-SSA1-2012) and international (FDA: CFR—Code of Federal Regulations Title 21, part 640) standards. Informed consent was given by all the participants before this study. Ethical approval (CI 2023-47) was provided by the General Hospital of Hermosillo, Sonora, Mexico. For the assay, a suspension of erythrocytes was elaborated with PBS (pH 7.4, 2%). Erythrocyte hemolysis was induced with AAPH generating reactive oxygen species, principally peroxyl radicals, as follows. A combination of erythrocytes (100 μL), PBS (100 μL), and AAPH (0.1085 g/mL, pH 7.4) (100 μL) was prepared and incubated at 37 °C, whilst being stirring at 30 rpm, in darkness, for 3 h. Subsequently, 1 mL of PBS was incorporated into the mixture and centrifuged at 1500 rpm for 10 min. The absorbance of the supernatant was read at λ = 540 nm. To determine the protective effect on the erythrocytes of the sample, a combination of erythrocytes (100 μL), the sample (100 μL), and AAPH (0.1085 g/mL, pH 7.4) (100 μL) was prepared like the procedure explained above. The results were expressed as a percentage of hemolysis inhibition (PHI), and the result was calculated employing Equation (16):

(16) $$PHI\left(\%\right)=\frac{AHI-AS}{AHI}\times 100$$

where AHI was defined as the absorbance of hemolysis induced by AAPH, while AS was defined as the absorbance of the sample (λ = 540 nm both).

### 4.18. Statistical Analysis

The experimental design was completely randomized. The data were subjected to an analysis of variance. The means analysis was performed using Fischer’s least significant difference or multiple range test (least significant difference—LSD). Differences of less than 0.05 (*p* < 0.05) were considered significant. Statgraphics Centurion XV software (version 16.2) was used.

## 5. Conclusions

This present study successfully formed an inclusion complex using β-C as the active compound (the guest molecule) and β-CD (the host molecule). The total release of β-C was found at 168 h. The release kinetics data, in synergy with the physical mechanisms involved in the experimental release, demonstrate that mass transport is given by diffusion. The sorption kinetics test provided interaction data between the adsorbate and absorbent when they reach equilibrium, indicating that the inclusion complex can be used for long periods. This advantage could apply to avoiding food oxidation during the storage of, for example, fresh-cut fruits. The parameters that describe the rehydration process were found, and intraparticle diffusion is the only step that controls the adsorption rate. The sorption studies showed that the β-C/β-CD inclusion complex had a reduction in the amount of water that pure β-CD can absorb. The antioxidant capacity is maintained in β-C/β-CD, stabilizing the complex formed. Furthermore, the β-C/β-CD inclusion complex presented a protective effect against the oxidative damage caused by the radicals in human erythrocytes. In this way, chronic degenerative diseases can be avoided, although more studies are still needed.

## Figures and Tables

**Table 1:** Yield, entrapment efficiency (EE%), and loading efficiency (LE%) of inclusion β-C/β-CD complex.

β-C/β-CD Ratio (%*w*/*w*)	Initial Weight β-C (g)	Initial Weight β-CD (g)	Initial Weight β-C/β-CD (g)	Recovery β-C/β-CD (g)	Yield (%) ^A^	Weight of Encapsulated β-C (g) ^A^	EE (%) ^A^	LE (%) ^A^
40:60	0.4	0.6	1.0	0.941 ± 0.012	94.10 ± 1.21	0.3298 ± 0.03	82.47 ± 0.40	35 ± 0.24

^A^ Mean ± standard deviation (SD) of three independent experiments.

**Table 2:** Values of R^2^ for each kinetic release model and the transport exponent (n) of the β-C:β-CD inclusion complex at temperatures of 8 and 25 °C.

Temperature	Zero Order	First Order	Higuchi	Korsmeyer–Peppas	
	R^2^	R^2^	R^2^	R^2^	n
8 °C	0.8079	0.4052	0.9544	0.9909	0.349
25 °C	0.8070	0.2972	0.9332	0.9887	0.264
37 °C	0.8069	0.2720	0.9195	0.9895	0.230

**Table 3:** The values of R^2^ for each release kinetic model of the β-C and β-C/β-CD inclusion complex at 8 °C and the parameters of Peleg’s model for moisture sorption. The constant K_1_ (min%-1) provides information on the water absorption rate, especially during the initial stage of the process discussed (R_0_). The constant K_2_ (%-1) predicts the maximum water absorption capacity (M_∞_).

Molecule	Pseudo-First Order	Pseudo-Second Order	Peleg’s Model					
	R^2^	R^2^	R^2^	K_1_	K_2_	R_0_	M_∞_	R^2^
*β*-CD	0.9191	0.8802	0.9507	17.412	0.079	13.825	33.825	0.9958
*β*-C:*β*-CD	0.9745	0.9142	0.9778	9.245	0.072	12.690	32.690	0.9999

**Table 4:** Antioxidant activity of β-C and β-C/β-CD inclusion complex.

Sample	Concentration (mg/mL)	ABTS (% Inhibition)	DPPH (% Inhibition)	FRAP (μM TE/g)
β-CD	0.5	0	0	0
β-C	0.01	3.21 ± 0.31 ^f^	1.65 ± 0.33 ^f^	0.36 ± 0.07 ^f^
	0.03	5.65 ± 0.53 ^e^	3.75 ±0.09 ^e^	0.83 ± 0.02 ^e^
	0.05	8.21 ± 0.31 ^d^	5.99 ± 0.53 ^d^	1.26 ± 0.11 ^d^
	0.10	14.22 ± 1.14 ^c^	11.64 ± 2.13 ^c^	3.23 ± 0.71 ^c^
	0.30	22.54 ± 2.03 ^b^	18.98 ± 1.45 ^b^	5.77 ±0.33 ^b^
	0.50	35.64 ± 1.92 ^a^	22.63 ± 0.98 ^a^	9.12 ± 0.67 ^a^
β-C/βCD *	0.03	2.74 ± 0.01 ^f^	1.35 ± 0.37 ^f^	0.47 ± 0.53 ^e^
	0.09	4.53 ± 0.03 ^e^	3.01 ± 0.82 ^e^	0.77 ±0.04 ^e^
	0.15	8.63 ± 0.42 ^d^	5.35 ± 0.35 ^d^	1.08 ± 0.53 ^d^
	0.30	13.67 ± 1.13 ^c^	10.41 ± 1.13 ^c^	2.99 ± 0.32 ^c^
	0.90	23.38 ± 2.35 ^b^	17.88 ± 2.28 ^b^	5.01 ± 0.91 ^b^
	1.50	34.09 ± 1.15 ^a^	21.73 ± 2.84 ^a^	8.85 ±0.74 ^a^
Ascorbic acid	0.01	25.76 ± 0.22 ^f^	13.25 ± 1.5 ^f^	1.21 ± 1.02 ^e^
	0.03	31.06 ±3.14 ^e^	22.76 ± 2.54 ^e^	2.75 ± 1.65 ^e^
	0.05	45.23 ± 0.12 ^d^	37.21 ± 3.5 ^d^	3.21 ± 2.32 ^d^
	0.10	57.87 ± 3.11 ^c^	41.85 ± 1.11 ^c^	5.86 ± 3.01 ^c^
	0.30	66.98 ± 2.07 ^b^	56.73 ± 2.28 ^b^	7.11 ± 2.26 ^b^
	0.50	74.33 ± 3.35 ^a^	61.93 ± 2.08 ^a^	8.34 ± 0.93 ^a^
Solvent	0.50	0.05 ± 0.001	0.93 ± 0.03	0.01 ± 0.005

* The calculated amount of β-C/βCD (0.03–1.5 mg/mL) to correspond to the amount of complexed β-C (0.01–0.5 mg/mL). The values are shown as the mean ± standard deviation of triplicate determinations. The different lowercase letters by column indicate a significant difference (*p* < 0.05). The controls in the DPPH, ABTS, and FRAP compound determinations were the reagents with the solvent of the samples, and a positive control was ascorbic acid. μM TE/g = micromoles of Trolox Equivalents by a gram of sample.

**Table 5:** Percentage of inhibition of hemolysis of β-C, β-CD, β-C/β-CD, ascorbic acid, and AAPH.

	Concentration (mg/mL)	% Inhibition of Hemolysis
β-CD	0.5	0
AAPH	0.5	0
β-C	0.01	9.01 ± 0.93 ^e^
	0.03	10.05 ± 1.58 ^e^
	0.05	21.76 ± 2.31 ^d^
	0.10	27.53 ± 1.02 ^c^
	0.30	36.33 ± 1.55 ^b^
	0.50	51.33 ± 2.04 ^a^
β-C/βCD *	0.03	8.95 ± 1.11 ^f^
	0.09	14.87 ± 1.04 ^e^
	0.15	25.63 ± 1.23 ^d^
	0.30	32.01 ± 2.97 ^c^
	0.90	46.76 ± 1.73 ^b^
	1.50	64.09 ± 1.66 ^a^
Ascorbic acid	0.01	5.06 ± 0.88 ^f^
	0.03	13.06 ±1.63 ^e^
	0.05	35.23 ± 0.12 ^d^
	0.10	49.92 ± 2.09 ^c^
	0.30	58.21 ± 3.11 ^b^
	0.50	70.89 ± 2.05 ^a^
Solvent	0.50 ^a^	0.00 ± 0.000

* The calculated amount of β-C/βCD (0.03–1.5 mg/mL) to correspond to the amount of complexed β-C (0.01–0.5 mg/mL). The values are shown as the mean ± standard deviation of triplicate determinations. The different lowercase letters by column indicate a significant difference (*p* < 0.05). The controls in the DPPH, ABTS, and FRAP compound determinations were the reagents with the solvent of the samples, AAPH, and ascorbic acid. μM TE/g = micromoles of Trolox Equivalents by a gram of sample.

## Data Availability

The original contribution data presented in this research are included in this article; further inquiries can be directed to the corresponding authors. The data are not publicly available since the author of the correspondence keeps control of its diffusion according to the regulations of the University of Sonora. However, the information can be requested without problem by the people who require it.

## Abbreviations

The following abbreviations are used in this manuscript:

AAPH	2,2′-Azobis(2-amidinopropane) dihydrochloride
ABTS	2,2′-azinobis(3-ethylbenzothiazoline
AD	Average diameter
AHI	Absorbance of hemolysis induced
AS	Absorbance of the sample
CD	Cyclodextrin
DPPH	2,2-diphenyl-1-picrilhydrazil
DSC	Differential scanning calorimetry
EE	Entrapment efficiency
FRAP	Ferric reducing antioxidant power
FT-IR	Fourier transform infrared spectroscopy
LE	Loading efficiency
PBS	Phosphate buffer saline
PDI	Polydispersity index
RH	Relative humidity
SD	Standard deviation
SEM	Scanning electron microscopy
TE	Trolox Equivalents
TPTZ	2,4,6-tripyridyl-s-triazine
XRD	X-ray diffraction
ZP	Zeta potential
β-C	β-Carotene
β-C/β-CD	β-Carotene in β-cyclodextrin inclusion complexes
β-CD	β-cyclodextrin
